# Supplementary material for: Translational contributions to tissue specificity in rhythmic and constitutive gene expression
Source: Genome Biol. 2017 Jun 16;18:116. doi: 10.1186/s13059-017-1222-2 (PMC5473967; doi:10.1186/s13059-017-1222-2)
Supplement: Supplementary file 7 — Expression plots for kidney and liver for the 178 common rhythmic genes of Fig. 3c. (ZIP 3338.28 kb) [file 13059_2017_1222_MOESM7_ESM.zip › set_D_shared(178)/Mt1_kidney_set_D.pdf]

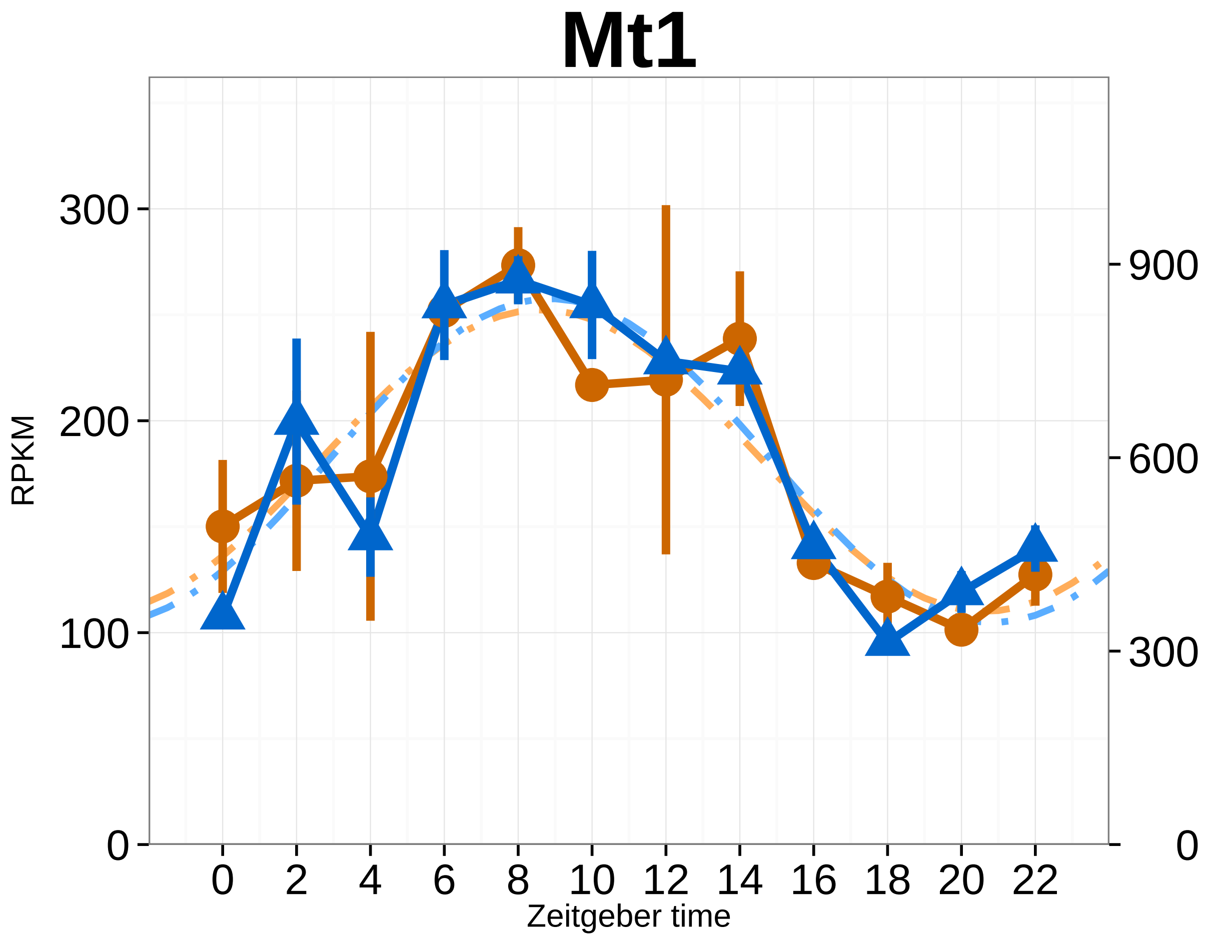

Mt1
RPKM
300
200
100
0
900
600
300
0
0
2
4
6
8
10
12
14
16
18
20
22
Zeitgeber time

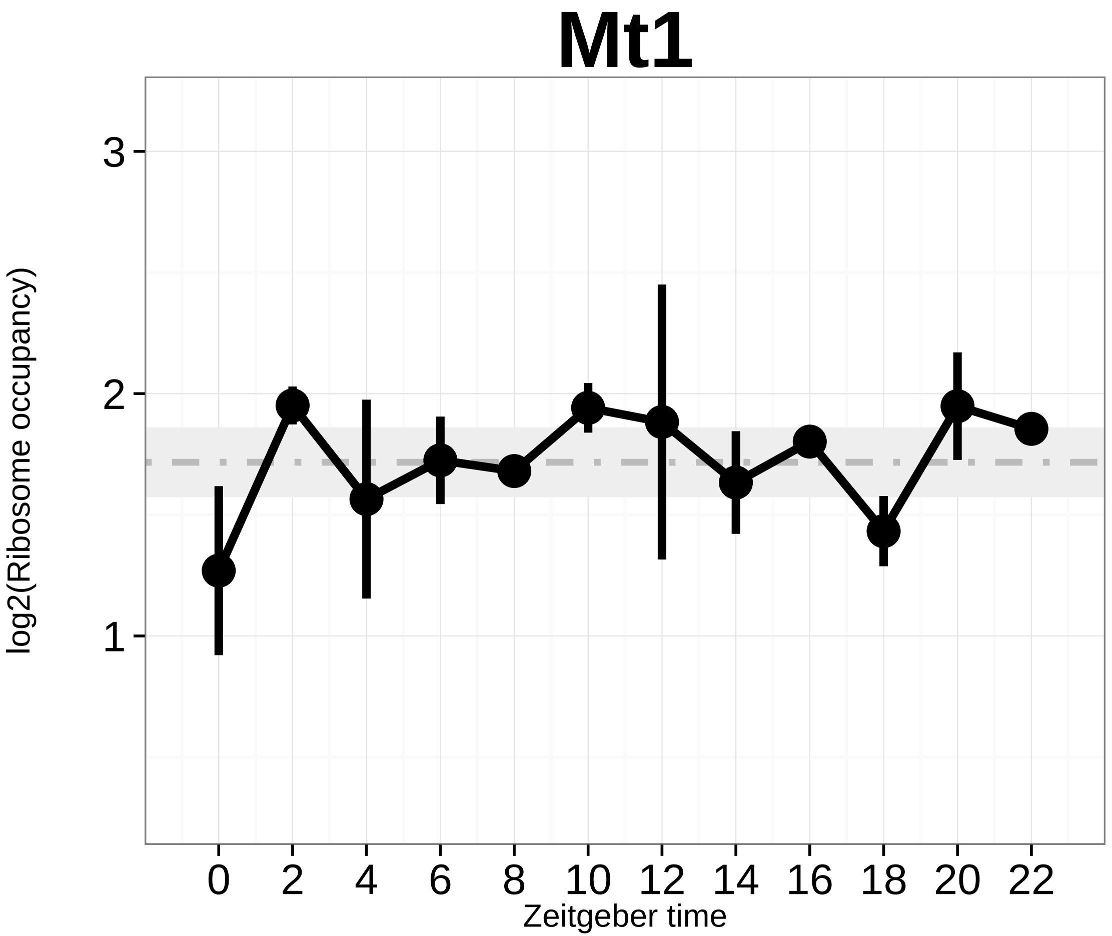

Mt1
log2(Ribosome occupancy)
3
2
1
0
2
4
6
8
10
12
14
16
18
20
22
Zeitgeber time
